# Serotype Distribution and Antimicrobial Resistance of *Salmonella* Isolates from Poultry Sources in China

**DOI:** 10.3390/antibiotics13100959

**Published:** 2024-10-11

**Authors:** Chu Wang, Xianwen Wang, Juyuan Hao, He Kong, Liyuan Zhao, Mingzhen Li, Ming Zou, Gang Liu

**Affiliations:** 1 College of Veterinary Medicine, Qingdao Agricultural University, Qingdao 266109, China; wangchu@stu.qau.edu.cn (C.W.); wxw@stu.qau.edu.cn (X.W.); haojuyuan@stu.qau.edu.cn (J.H.); konghe@stu.qau.edu.cn (H.K.); zhaoliyuan@stu.qau.edu.cn (L.Z.); mzou@qau.edu.cn (M.Z.); 2 Shandong Provincial Center for Animal Disease Control (Shandong Provincial Center for Zoonoses Epidemiology Investigation and Surveillance), Jinan 250100, China; zh990923@126.com

**Keywords:** *Salmonella*, broilers, laying hens, ARGs, serotypes, antimicrobial resistance

## Abstract

Background: *Salmonella* is an important zoonotic pathogen, of which poultry products are important reservoirs. This study analyzed the prevalence, antimicrobial resistance, and characterization of *Salmonella* from broiler and laying hen sources in China. Methods: A total of 138 (12.27%) strains of *Salmonella* were isolated from 1125 samples from broiler slaughterhouses (20.66%, 44/213), broiler farms (18.21%, 55/302), and laying hen farms (6.39%, 39/610). Multiplex PCR was used to identify the serotypes. Antibiotic susceptibility testing to a set of 21 antibiotics was performed and all strains were screened by PCR for 24 selected antimicrobial resistance genes (ARGs). In addition, 24 strains of *Salmonella* were screened out by whole-genome sequencing together with 65 released *Salmonella* genomes to evaluate phylogenetic characteristics, multilocus sequence typing (MLST), and plasmid carriage percentages. Results: A total of 11 different serotypes were identified, with the dominance of *S*. Enteritidis (43/138, 31.16%), *S*. Newport (30/138, 21.74%), and *S*. Indiana (19/138, 13.77%). The results showed that *S*. Enteritidis (34.34%, 34/99) and *S*. Newport (51.28%, 20/39) were the dominant serotypes of isolates from broilers and laying hens, respectively. The 138 isolates showed the highest resistance to sulfisoxazole (SXZ, 100%), nalidixic acid (NAL, 54.35%), tetracycline (TET, 47.83%), streptomycin (STR, 39.86%), ampicillin (AMP, 39.13%), and chloramphenicol (CHL, 30.43%), while all the strains were sensitive to both tigacycline (TIG) and colistin (COL). A total of 45.65% (63/138) of the isolates were multidrug-resistant (MDR) strains, and most of them (61/63, 96.83%) were from broiler sources. The results of PCR assays revealed that 63.77% of the isolates were carrying the quinolone resistance gene *qnrD*, followed by *gyrB* (58.70%) and the trimethoprim resistance gene *dfrA12* (52.17%). Moreover, a total of thirty-four ARGs, eighty-nine virulence genes, and eight plasmid replicons were detected in the twenty-four screened *Salmonella* strains, among which *S*. Indiana was detected to carry the most ARGs and the fewest plasmid replicons and virulence genes compared to the other serotypes. Conclusions: This study revealed a high percentage of multidrug-resistant *Salmonella* from poultry sources, stressing the importance of continuous monitoring of *Salmonella* serotypes and antimicrobial resistance in the poultry chain, and emergency strategies should be implemented to address this problem.

## 1. Introduction

*Salmonella* is a common foodborne pathogen that causes salmonellosis, a global gastrointestinal infection [1]. The typical symptoms of salmonellosis include diarrhea, fever, abdominal pain, and vomiting, often following the ingestion of contaminated food or water [2]. *Salmonella* can survive in a variety of animal hosts, including poultry, livestock, and pets, which increases the risk of human infection with *Salmonella* [3,4,5]. *Salmonella* derived from chicken and other poultry products is one of the main causes of contamination of eggs and chicken products, posing a serious threat to food safety and public health. According to the U.S. Department of Agriculture (USDA), 37% of chickens tested positive for *Salmonella*, while 50–100% of other poultry and eggs have also been shown to carry *Salmonella* [6]. In China, approximately 70–80% of foodborne bacterial outbreaks can be attributed to *Salmonella* infection [7].

As an important foodborne pathogen, the increasing drug resistance of *Salmonella* has attracted global attention [8,9]. In recent years, *Salmonella* strains in chicken and other poultry products in Asian counties have become increasingly resistant to most of the commonly used antibiotics, such as fluoroquinolones, β-lactams, tetracyclines, and aminoglycosides [10]. High resistance to penicillin, tetracyclines, and sulfonamides in *Salmonella* strains isolated from chickens was observed, with 43.52% of the isolates being multidrug-resistant bacteria with complex resistance profiles in Anhui Province, China [11]. The increase in drug resistance reduces the options for effective drugs to treat *Salmonella* infections, leading to treatment failure and prolonged infection duration, seriously affecting the economic benefits of farmers [12,13,14].

To date, more than 2600 *Salmonella* enterica serovars have been identified, and different *Salmonella* serotypes exhibit significant differences in drug resistance, which is of great significance for the treatment and control of *Salmonella* infections [15,16]. For example, *S.* Enteritidis and *S.* Typhimurium are the main serotypes of human infection, and they exhibit high resistance to multiple antibiotics [17]. In addition, serotypes such as *S.* Indiana and *S.* Enteritidis have also been reported to be resistant to specific antibiotics [7]. Surveillance data worldwide have shown that drug resistance in *Salmonella* is constantly changing, and new drug-resistant strains and resistance patterns continue to emerge [18].

During the last decade, several recurrent outbreaks of avian salmonellosis have been reported in China, but the specific causal agent remains unclear. Most studies about the prevalence and drug resistance of *Salmonella* have been limited to a certain region, animal source, or in partial processing stages in the slaughtering line, and large-scale horizontal research is lacking [19,20,21]. This study characterized relevant isolates from broilers and laying hens, and investigated the phenotypic and genotypic diversity of *Salmonella* strains from different sources. In addition, we also conducted comparisons and genomic epidemiological analyses with existing relevant data in NCBI based on their serotypes and sources. Our findings contribute to understanding the differences between the current status of *Salmonella* epidemics in China and the world, and to provide a research basis for the prevention and control of this foodborne pathogen.

## 2. Results

### 2.1. Isolation and Serotypes

**Figure 1:**
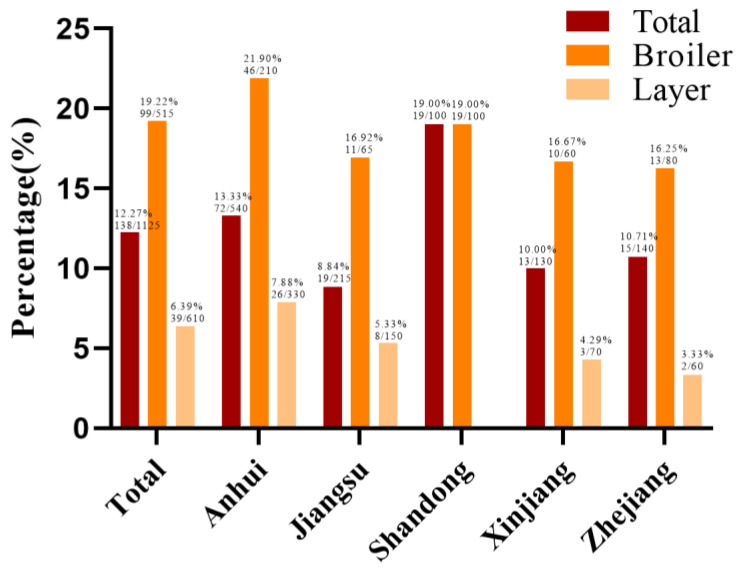
Area, source, and overall isolation percentages of *Salmonella*. The Y-axis represents the positive percentage of *Salmonella*, and the X-axis represents the sampling area. The three different-colored columns represent the overall, broiler source, and laying hen source. There were no laying hen samples from Shandong Province, so the overall positivity percentage was consistent with the positivity percentage of the broiler samples.

In this study, a total of 138 *Salmonella* strains were identified from 1125 samples, with a positive percentage of 12.27% (138/1125). Among them, 19.22% (99/515) of the strains were derived from broiler sources, which is obviously higher than that of laying hens (39/610, 6.39%) (Figure 1). Among the broiler samples, the isolation percentages of the slaughterhouses and farms were 20.66% (44/213) and 18.21% (55/302), respectively. The sample from Shandong Province had the highest percentage of *Salmonella* (19.00%, 19/100), followed by Anhui Province (13.33%, 72/540), Zhejiang Province (10.71%, 15/140), Xinjiang Province (10.00%, 13/130), and Jiangsu Province (8.84%, 19/215) (Figure 1).

**Figure 2:**
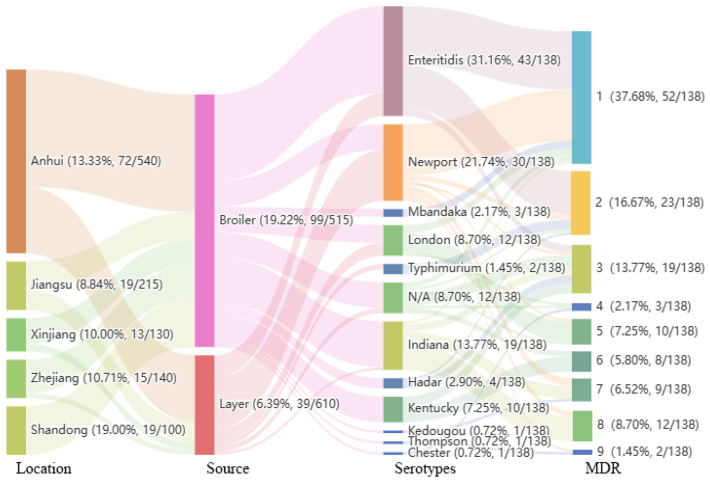
Sankey diagram of the regional distribution, source, serotype, and MDR index of all strains. The diameter of the line is proportional to the number of isolates from a given source, which is also shown in parentheses on the right. MDR denotes multidrug resistance.

Overall, a total of 11 different serotypes were detected, with *S*. Enteritidis (31.16%, 43/138) being the main dominant serotype, followed by S. Newport (21.74%, 30/138,) and *S*. Indiana (13.77%, 19/138). Among the remaining *Salmonella* strains, 8.70% (12/138) were *S*. London, 7.25% (10/138) were *S*. Kentucky, 2.90% (4/138) were *S*. Hadar, 2.17% (3/138) were *S*. Mbandaka, 1.45% (2/138) were *S*. Typhimurium, *S*. Chester, *S*. Thompson, and *S*. Kedougou each accounted for 0.72% (1/138), while 8.70% (12/138) of the *Salmonella* strains were of unknown serotype (Figure 2). Among the *Salmonella* isolates from the broiler samples, *S*. Enteritidis (34.34%, 34/99) was the main dominant serotype, followed by *S*. Indiana (18.18%, 18/99), *S*. Kentucky (10.10%, 10/99), and *S*. Newport (10.10%, 10/99). For laying hens, *S*. Newport (51.28%, 20/39) was the main dominant serotype, followed by *S*. Enteritidis (23.08%, 9/39) and *S.* London (12.82%, 5/39).

### 2.2. Antibiotic Resistance and MDR Profiles

**Figure 3:**
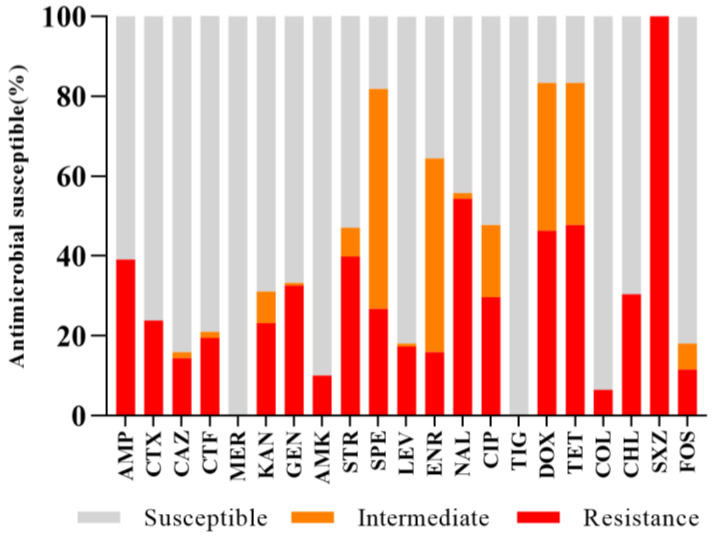
Antibiotic susceptibility pattern of *Salmonella* isolates. The X-axis represents the antibiotics used, and the Y-axis represents the proportion of strains with different sensitivities to the drugs. Red represents resistance, orange represents intermediate, and gray represents susceptible.

For the antimicrobial susceptibility test performed on the *Salmonella* strains isolated in this study, the results of the antibiotic susceptibility test were divided into three categories: resistant, intermediate, and susceptible, according to the standards specified by the CLSI (Figure 3). Antimicrobial susceptibility testing revealed that the obtained *Salmonella* isolates had the highest resistance to SXZ (100%, 138/138), followed by NAL (54.35%, 75/138), TET (47.83%, 66/138), DOX (46.38%, 64/138), STR (39.86%, 55/138), AMP (39.13%, 54/138), GEN (45/138, 32.61%), and CHL (42/138, 30.43%), while only 6.52% (9/138) were resistant to COL (Table 1). Importantly, resistance to TIG and MER was not detected in any of the isolates. Most of the antibiotics had high MIC_90_ levels, especially SXZ, NAL, AMP, CTX, GEN, STR, SPE, CHL, and FOS, the MIC_90_ values of which were higher than the highest tested concentrations (Table 1). Among the 138 isolates, 62.32% (86/138) were resistant to at least two classes of antibiotics, while 45.65% (63/138) were MDR strains which were resistant to at least three classes of antibiotics (Figure 2). Most of the MDR strains were from broiler sources (96.83%, 61/63) and only a few were from laying hen sources (3.17%, 2/63).

### 2.3. Analysis of Antibiotic Resistance Genes

**Figure 4:**
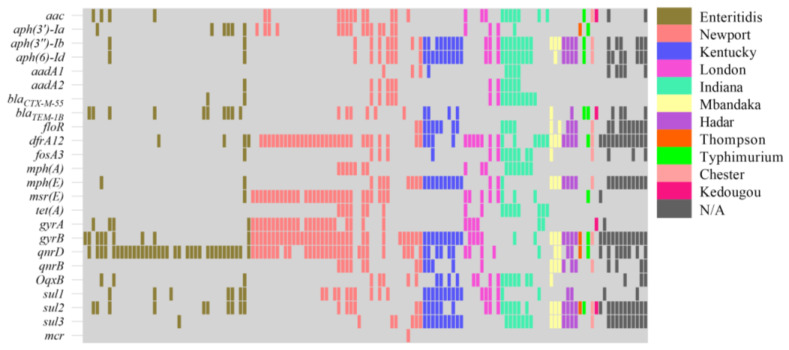
Heatmap showing the AMR gene profiles identified in this study. Different groups of serotype strains are color-coded. The heatmap shows the profile of drug resistance genes detected in the studied isolates. The Y-axis shows the drug resistance gene detected, and the X-axis shows the serotype to which the detected strain belongs. Light gray, negative.

The 24 ARGs in 10 categories were identified using PCR for these 138 isolates. The quinolone resistance genes *qnrD* (63.77%, 88/138) and *gyrB* (58.70%, 81/138) were the two genes with the highest detection percentages, followed by trimethoprim *dfrA12* (52.17%), *gyrA* (26.09%), *qnrB* (17.39%), and *oqxB* (19.57%) (Figure 4). For the β-lactam ARGs, *bla*_CTX-M-55_ and *bla*_TEM-1B_ were detected in 18 (13.04%) and 28 (21.01%) strains, respectively. In addition, the aminoglycoside ARGs *aac* (22.46%), *aph*(*3′*)-*Ia* (19.57%), *aph*(*3″*)-*Ib* (32.61%), *aph*(*6*)-*Id* (31.88%), *aadA1* (9.42%), *aadA2* (9.42%); the macrolide ARGs *mphA* (12.32%), *mphE* (29.71%), and *msrE* (29.71%), the tetracycline ARG *tetA* (12.32%); the sulfonamides ARGs *sul1* (26.81%), *sul2* (38.41%), and *sul3* (30.43%); the chloramphenicol ARG *floR* (20.29%); and the fosfomycin ARG *fosA3* (13.77%) were identified in this study. Furthermore, the carriage of ARGs varied among the different serotype isolates, and compared to other serotypes, *S*. Chester, *S*. Hardar, *S*. Mbandake, and *S*. Kentucky carried the most ARGs, while *S*. Enteritidis carried fewer (Figure 4).

### 2.4. WGS Analysis

**Figure 5:**
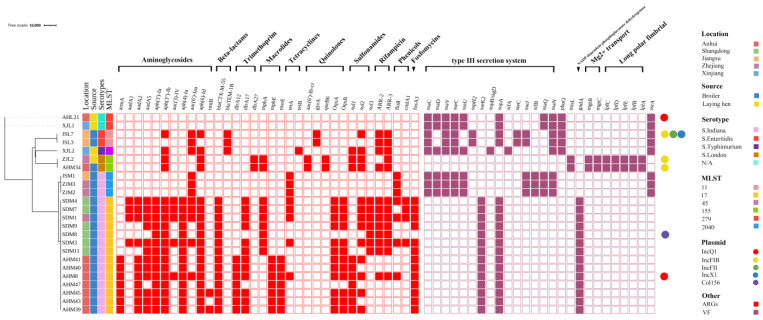
Phylogenetic structure, region, sample source, serotype, MLST, antibiotic resistance genotype, virulence genotype, and replicon typing of 24 *Salmonella* strains. Hollow cells do not carry the relevant gene. Circles, replicon typing.

Based on the MDR profiles and different regional sources, 24 *Salmonella* strains were selected for whole-genome sequencing. In silico MLST analysis revealed that fourteen out of the seventeen *S*. Indiana isolates had an MLST classification of ST17, and the remaining three isolates had an MLST classification of ST2040 (Figure 5). JSL3 and JSL7 were identified as *S*. Enteritidis, and their MLSTs were both ST11. AHM34 and ZJL2 were identified as *S*. London, and their MLST typing was both ST155. XJL2 was identified as *S*. Typhimurium, and its MLST type was ST34. The serotypes of XJL1 and AHL21 appear to be unknown serotypes, and their MLST types were both ST279 (Figure 5).

A total of 34 ARGs were detected from the sequenced strains, and *S*. Indiana isolates carried more resistance genes than isolates of other serotypes, including the ARGs of aminoglycosides, quinolones, β-lactams, and tetracyclines (Figure 5). Through analysis, it was found that only a few of these *S*. Indiana strains contained plasmids and most of these genes were located on chromosomes, indicating that the probability of antimicrobial resistance genes being transferred and spread to other bacteria was low. According to phylogenetic analysis, the genetic distance of the 17 *S*. Indiana isolates in this study was relatively close (Figure 5), indicating that there may be spread across the three provinces of Anhui, Zhejiang, and Shandong.

A total of 89 virulence genes were identified using BLASTn searches against the VFDB with an E-value < 1.0 × 10^−6^, of which 26 virulence genes with an E-value of 0 were further analyzed (Figure 5). Among them, fifteen virulence genes associated with the type III secretion system, such as *sifAB* (16.67%, 4/24), *sopAB* (79.17%, 19/24), *sseK2* (58.33%, 14/24), *ssaC* (33.33%, 8/24), *sseC* (33.33%, 8/24), *ssaN* (33.33%, 8/24), *ssaV* (33.33%, 8/24), *ssaD* (25.00%, 6/24), *ssaU* (5.00%, 6/24), *ssaQ* (20.83%, 5/24), *sseJ* (20.83%, 5/24), *sspH2* (8.33%, 2/24), and *steC* (8.33%, 2/24); five genes associated with long polar fimbrial proteins, including *lpfABCDE* (41.67%, 10/24); and two genes *mgtB* (8.33%, 2/24) and *mgtC* (8.33%, 2/24) associated with Mg^2+^- transport were identified. Other virulence genes included *gndA* (58.33%, 14/24), *phoQ* (20.83%, 5/24), *misL* (8.33%, 2/24), and *ssrA* (33.33%, 8/24) (Figure 5). A total of eight plasmid replicon types were detected, including IncQ1 (8.33%, 2/24), IncFIB (12.50%, 3/24), IncFII (4.17%, 1/24), IncX1 (4.17%, 1/24), Col156 (4.17%, 1/24), IncI2 (4.17%, 1/24), IncHI2 (4.17%, 1/24), and Inc I1-1 (4.17%, 1/24) (Figure 5). Notably, the diversity of plasmid types in *S*. Enteritidis JSL7 was higher than that in the other tested strains, and the plasmid carrying percentage of *S*. Indiana was significantly lower than that of other serotypes of *Salmonella*.

**Figure 6:**
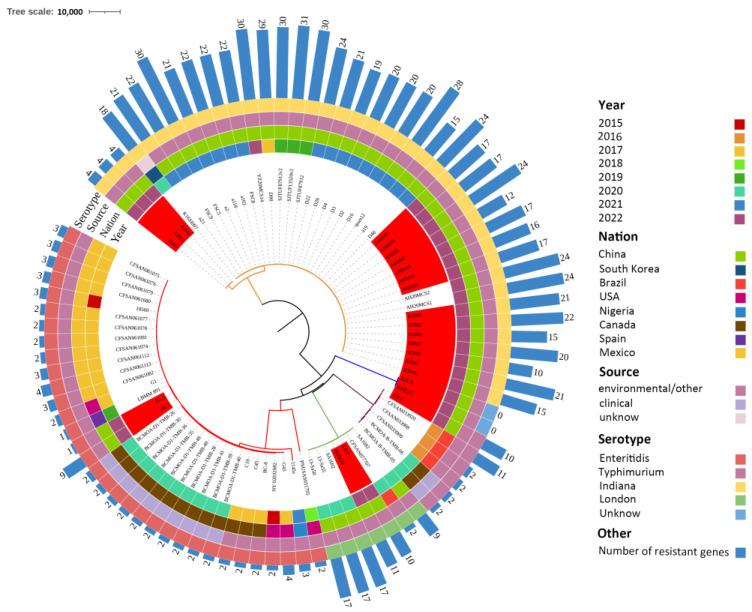
Phylogenetic tree of strains of the same serotype and origin from the NCBI database worldwide. Different-colored branches represent closer relationships. The red highlights are the strains used in this study. The circles from inside to outside represent the regional year of the strain (circle 1), the geographical origin of the strain (circle 2), the sample source of the strain (circle 3), the serotype of the strain (circle 4), and the number of drug resistance genes carried by the strain (bar graph).

According to the comprehensive analysis of 65 complete *Salmonella* genomes with a longitudinal span from the NCBI GenBank database and 24 genomes from our study, *S*. Indiana carried the most ARGs, followed by *S*. London and *S*. Typhimurium, while *S*. Enteritidis carried fewer ARGs (Figure 6). Among the different years, the number of ARGs carried was not significantly different (*p* = 0.1114), but a decreasing tread was observed (Figure 6). Phylogenetic analysis revealed that *S*. Indiana was closely related to the samples isolated from Anhui Province (Figure 6). Notably, from the perspective of regional distribution, in addition to unique strains, strains of the same serotype are highly homologous. Among the seven strains of *S*. London, the strain isolated from Brazil was closely related to the strains isolated from China. The *S*. Typhimurium strains in the present study were closely related to three strains isolated from Brazilian environmental samples and two strains isolated from Canada. The two strains of *S*. Enteritidis identified in this study were closely related to strains from Mexico (*n* = 12 strains), the United States (*n* = 4), Spain (*n* = 1), Nigeria (*n* = 1), and Canada (*n* = 13) (Figure 6).

## 3. Discussion

In this study, the percentage of *Salmonella* isolated from all samples in poultry sources collected from five provinces in China was 12.27%, which is consistent with previous reports of chickens (12.54%, 105/837) in central China, but slightly higher than the percentage of *Salmonella* isolated from broiler farms in Southeast Asia, as well as the Zhejiang and Fujian regions [22,23,24]. In this study, the percentage of *Salmonella*-positive chickens from broiler sources (19.22%) was slightly lower than that reported by commercial chicken farms in Henan, China (23.56%, 504/2139) [25] and higher than that reported in Qingdao, Shandong (14.98%, 172/1148) [26], but much higher than that reported from laying hen sources (6.39%). These findings are consistent with previous studies showing that chicken production is an important transmission route between *Salmonella* and food [27,28], and suggest that timely detection and isolation of suspected diseased chickens during broiler farming can be effective in interrupting the transmission of salmonellosis. In addition, the detection percentage of *Salmonella* isolates in Anhui Province was the highest in our study, although a previous study on the prevalence of *Salmonella* from chickens in Anhui Province showed that the positive isolation percentage of *Salmonella* from chicken samples was only 5.66% (108/1908), lower than the 13.33% (72/540) of our study results; however, in their study, 90% (45/50) of the samples came from laying hens, and only 10% (5/50) came from broiler sources.

The increasing rate of antibiotic resistance in *Salmonella* poses a significant global concern, and an improved understanding of the distribution of antibiotic resistance patterns in *Salmonella* is essential for choosing the suitable antibiotic for the treatment of infections. We found that *Salmonella* showed the highest resistance to SXZ and NAL, similar to the results of other studies [29,30], which may indicate the extensive use of these antimicrobial drugs in the poultry industry for rapid growth and disease prevention. In addition, we detected a high percentage of resistance to TET and AMP, which is consistent with the fact that these two antibiotics are high-frequency antibiotics used in the animal farming process [31]. Moreover, we found that the *Salmonella* isolates in this study were mainly resistant to CAZ, AMK, and FOS at equally high levels, whereas they were susceptible to MER and TIG, possibly because of the limited or restricted use of these antibiotics in the study area. In addition, 45.65% of the strains in this study were MDR to three or more drugs, with *Salmonella* of broiler origin accounting for the vast majority (96.83%). Although the percentage of MDR in *Salmonella* of broiler origin was lower than that reported in previous studies [32,33,34,35], MDR *Salmonella* are still a significant concern in poultry production that need to be monitored continuously. Among the MDR bacteria in this study, the most serious MDR was nine, and such serious resistance has rarely been reported before. Among the five provinces, the multi-resistant percentage of strains from Shandong was the most severe, which was similar to previous reports [36,37], but higher than that reported by Zhao [38]. Notably, among the MDR strains, at least seven were found, which might be related to the distribution of serotypes. Unlike other studies [17,39], all *S.* Enteritidis strains in this study exhibited low resistance proportions, which may be the result of limited antibiotic use.

Among the isolates, a total of 11 serotypes were identified, of which *S.* Enteritidis was the predominant serotype, followed by *S.* Newport and *S.* Indiana, which is consistent with the results of previous studies [7,40,41]. *S.* Enteritidis and *S.* Typhimurium are the most common serotypes that can cause infections in humans, leading to severe gastrointestinal illness [25], and the high positivity rate of *S.* Enteritidis in broiler sources may result in a high risk of disease. It is worth noting that the most common serotype of *Salmonella* in laying hens is *S.* Newport, which is one of the most common pathogens causing gastroenteritis in humans in the United States and can lead to severe invasive infections [42]. This finding suggests that although the positivity rate in laying hens is lower than that in broilers, it is still a risk factor that should not be ignored. According to data from China’s National Foodborne Disease Surveillance Network, the most prevalent serotypes of nontyphoid *Salmonella* infections in Zhejiang Province in the past decade (2010–2019) were *S.* Enteritidis and *S.* Typhimurium [43]. Our results suggest that the widespread distribution of foodborne *Salmonella* serotypes in chicken flocks may pose a threat to food safety, a conclusion also confirmed by the literature [44,45]. Differences in the distribution of *Salmonella* serotypes in different studies are related to regional differences and may also be related to the source and type of samples selected, such as *S.* Enteritidis and *S.* Newport, the most common serotypes of *Salmonella* isolated from broiler sources and laying hens, respectively [46]. Compared with the results of previous studies, there is an obvious upward trend [11], which is consistent with the findings of Victoria; however, a study in Virginia found the opposite [47,48]. The infection rates of *S.* Indiana and *S.* Newport were relatively high, but *S.* Typhimurium, which has been shown to be the dominant serotype of *Salmonella* in China in other studies, had only a low positive rate. Although some serotypes may not currently be dominant, their prevalence may change over time and they may become the predominant serotype in a given region due to the selective pressure of multiple antimicrobial agents [49]. In addition, the serotypes of *Salmonella* isolated from broiler samples were more diverse than those from laying hens, and some serotypes were identified only in broiler samples, such as *S.* Kentucky, which is similar to the results of a previous study [27], which may be attributed to the different management modes. However, it also reminds us that the biological control of the breeding process of broilers in China can refer to the measures taken in the case of laying hens.

In this study, a total of 24 ARGs were identified on 138 strains of *Salmonella*. By comparing the ARGs and drug-resistant phenotypes, it was found that the carriage of ARGs was not completely consistent with the drug-resistant phenotypes of the strains. The three situations of corresponding phenotypes and genes and missing genes or no phenotypes may be caused by the diversity of bacterial resistance mechanisms. This finding is consistent with previous studies reporting that it may be related to the selective silencing of certain genes under specific conditions [50]. In this study, *qnrD*, a plasmid-mediated quinolone resistance gene, was the most frequently detected resistance gene, suggesting the importance of strictly limiting the use of quinolones [51,52]. Among all identified ARGs, *gyrB* had a very high detection percentage (58.70%), but this percentage was significantly lower than the 75.51% reported in 2019 [53]. The *dfrA12* gene encoding dihydrofolate reductase also had a higher detection percentage than the other genes [54]. While all strains were highly resistant to SXZ, the sulfonamide resistance genes *sul1*, *sul2*, and *sul3* showed relatively low detection percentages, indicating that *Salmonella* may have new efflux pumps or resistance genes. This finding is similar to that of a previous report in which the resistance percentage to sulfamethoxazole was 97.3%, but no genes associated with sulfonamide resistance were detected [55].

According to the MLST typing results of the whole-gene-sequenced strains, all the ST17 strains were *S.* Indiana. Previous studies have shown that ST17 *S.* Indiana has existed in China for a long time and is widely distributed after experiencing large-scale population expansion [43,56]. In addition, our study revealed that the only *S.* Indiana isolate from South Korea was very close to our strain and may be the result of global spread. *S.* Indiana carries a large number of resistance genes because it carries a significantly higher proportion of integrons than the other serotypes [57]. *S.* Indiana from China generally carries a large number of ARGs but rarely contains plasmids, which may be related to the fact that most of the ARGs of these strains are located on the chromosome [4,58]. This finding is consistent with a study showing that there is a close association between specific serotypes and antibiotic resistance, and specific serotypes show a strong ability to integrate resistance genes [15]. Although almost all *S.* Indiana strains are resistant to tetracyclines, no genes mediating tetracycline resistance have been found in these strains, which is likely due to factors that lead to cross-resistance [59]. Our sequencing results also showed that for almost all ST17 *S.* Indiana strains, only two virulence genes, *ssek2* and *gndA*, were detected, which is also consistent with previous studies showing that the pathogenicity and lethality properties of this serotype strain were not high [58]. In other MLST types, including ST2040 *S.* Indiana, we detected long polar fimbria protein (*lpfABCDE*), type III secretion system effectors (*ssaC*, *sseC, sspH2*, *sopA*, *sifA*, *steC,* etc.), and the Mg^2+^ transport gene (*mgtBC*), among which the effector is a feature of the type III secretion system, indicating that the strain has the basis for exerting virulence [60]. In this study, a high degree of similarity in virulence genes carried by strains with the same MLST typing was found, which has also been demonstrated in a previous study [61], and it might be possible to derive the virulence genes of *Salmonella* by MLST typing, which can guide clinical dosing strategies.

## 4. Materials and Methods

### 4.1. Sample Collection

A total of 1125 samples were collected from Anhui (*n* = 540), Jiangsu (*n* = 215), Shandong (*n* = 100), Xinjiang (*n* = 130), and Zhejiang (*n* = 140) Provinces, including 213 carcass samples randomly collected from broiler slaughterhouses, 302 cloacal samples from broiler farms, and 610 cloacal samples from laying hen farms. All samples were collected using swabs with 1 mL of Liquid Amies Medium (Copan, Brescia, Italy), stored in cryogenic incubators and transferred to the laboratory for processing within 24 h.

### 4.2. Isolation and Identification of Bacteria

All the samples were pre-enriched in 9 mL of protein-buffered water (BPW; Landbridge, Beijing, China), and 0.1 mL of the pre-enriched mixture was added to 10 mL of selenite cystine enrichment solution (SC; Landbridge, Beijing, China) with shaking (180 rpm) at 37 °C for 12 h. Colony isolation was performed on xylose–lysine–deoxycholate agar (XLD; BD Biosciences, Franklin Lakes, NJ, USA) combined with *Salmonella* chromogenic medium (SCM; Oxoid, Basingstoke, UK), and culture at 37 °C for 18–24 h. Typical *Salmonella* colonies growing on plates that appeared black on the XLD plate and purple on the *Salmonella* chromogenic agar plate were identified. The positive *Salmonella* isolates were further confirmed by identifying the *Salmonella invA* gene [62], with *S.* Typhimurium ATCC 14028 serving as a positive control.

### 4.3. Serotyping

The serotype of *Salmonella* was detected by multiplex PCR according to methods described previously [63]. Briefly, the PCR mixture of the O antigen genes consisted of 12.5 μL of 2× Accurate Taq master mix (with dye), 0.5 μM of each set of forward and reverse primers, 2 μL of template DNA, and an appropriate amount of molecular-grade water to make a 25 μL reaction volume. The PCR conditions were as follows: incubation at 95 °C for 5 min, 30 cycles at 94 °C for 1 min, 55 °C for 1 min, and 72 °C for 1 min; and separation of the PCR products on a 2.0% agarose gel with TBE buffer at 100 V for 20 min. The sequenced strain ATCC 14028 was used as a positive control.

### 4.4. Identification of Minimum Inhibitory Concentrations (MICs)

The MICs were determined using the broth microdilution method according to the CLSI guidelines [64], which employed the following 21 antibiotics: ampicillin (AMP), ceftazidime (CAZ), cefotaxime (CTX), ceftiofur (CTF), kanamycin (KAN), meropenem (MER), amikacin (AMK), gentamicin (GEN), spectinomycin (SPE), streptomycin (STR), enrofloxacin (ENR), ciprofloxacin (CIP), levofloxacin (LEV), nalidixic acid (NAL), chloramphenicol (CHL), fosfomycin (FOS), tetracycline (TET), doxycycline (DOX), colistin (COL), sulfisoxazole (SXZ), and tigecycline (TIG). The drugs used in the experiment were purchased from Shanghai Macklin Biochemical Technology Co., Ltd., Shanghai, China, and the active ingredients or drug purity were ≥95%. *Escherichia coli* ATCC 25922 was used as the control strain.

### 4.5. Detection of Drug Resistance Genes

A total of 24 resistance genes of 10 major categories of antibiotics were screened by polymerase chain reaction (PCR), and primer sequences are shown in Supplementary Table S1. The primers were synthesized and provided by Sangon Biotech (Shanghai) Co., Ltd. (Shanghai, China). PCR was conducted in 25 μL reactions containing 12 μL of 2× Taq Master Mix, 0.5 μM each of the forward and reverse primers, 1 μL of template DNA, and 10 μL of molecular-grade water. The PCR conditions were as follows: incubation at 94 °C for 5 min, followed by 30 cycles of denaturation at 94 °C for 30 s, annealing at the appropriate temperature for 30 s, and extension at 72 °C for 1 min; and a final extension at 72 °C for 5 min. The PCR products were dyed by red gel and visualized under UV light (Bio-Rad, Hercules, CA, USA) after electrophoresis.

### 4.6. Whole-Genome Sequencing

According to the different resistance levels and sources of the strains, 24 strains were selected for whole-genome sequencing (Supplementary Table S2). The genomic DNA was extracted with a TIANamp Bacteria DNA Kit (Tiangen Biotech, Beijing, China), and WGS was completed using the Illumina NovaSeq 6000 sequencing platform (Novogene Co., Ltd., Beijing, China). The sequencing reads were quality trimmed using Trimmomatic v0.32 and assembled using SPAdes v3.9.0 (https://cge.food.dtu.dk/services/SPAdes/, accessed on 20 May 2024). ResFinder 4.3.3 (http://genepi.food.dtu.dk/resfinder, accessed on 22 May 2024) and PlasmidFinder 2.1 (https://cge.food.dtu.dk/services/PlasmidFinder/, accessed on 22 May 2024) were used to identify resistance genes and plasmids. SeqSero 1.2 (https://cge.food.dtu.dk/services/SeqSero/, accessed on 24 May 2024) and MLST 2.0 (https://cge.food.dtu.dk/services/MLST/, accessed on 25 May 2024) were used for the prediction of serotypes and multilocus sequence typing. The virulence islands contained in the sequenced strains were predicted by SPIFinder 2.0 (https://cge.food.dtu.dk/services/SPIFinder/, accessed on 4 June 2024). The virulence genes were investigated using the BLASTn method against the VFDB (http://www.mgc.ac.cn/VFs/search_VFs.htm, accessed on 6 June 2024) with an E-value < 1.0 × 10^−6^, and then genes with an E-value of 0 were selected for statistical analysis. In addition, a total of 65 *Salmonella* genomes (24 *S*. Indiana, 5 *S*. London, 5 *S*. Typhimurium, and 31 *S.* Enteritidis) with the same sample source were searched for comparison through the Isolates Browser on NCBI (https://www.ncbi.nlm.nih.gov/pathogens/isolates/, accessed on 20 June 2024). Neighbor-joining trees for phylogenetic analysis were drawn using NDtree 1.2 (https://cge.food.dtu.dk/services/NDtree/, accessed on 22 June 2024), and modified with interactive tree of life (https://itol.embl.de, accessed on 22 June 2024) [65].

### 4.7. Data Analysis and Usability

The analysis of the obtained results and the generation of figures were performed using GraphPad Prism (GraphPad, San Diego, CA, USA) version 7.03, and Student’s *t*-test with Welch’s correction was used in this study.

## 5. Conclusions

This study revealed that *Salmonella* contamination is common in poultry sources, and that the prevalence of *Salmonella* contamination in broiler sources was significantly higher than that in laying hen sources, indicating that chicken production is an important transmission route between *Salmonella* and food. In addition, the MDR *Salmonella* strains isolated in this study pose a serious risk to food safety and endanger public health. Therefore, biological control in broiler farming should be increased and prevention strategies should be strictly implemented. It is necessary to implement stricter medication management systems to minimize the risk of transmission of antibiotic resistance.

## Figures and Tables

**Table 1:** MIC_50_, MIC_90_, and AMR of all strains to the tested antibiotics in this study.

Antibiotic Category	Antibiotics	MIC Value (mg/L)		No. of Resistant Isolates (%)
		MIC_50_	MIC_90_	
β-lactams	AMP	2	>128	54 (39.13)
	CTX	0.03	>128	33 (23.91)
	CAZ	0.5	64	20 (14.49)
	CTF	0.5	>64	27 (19.57)
	MER	0.125	0.125	0
Aminoglycosides	KAN	2	>64	32 (23.19)
	GEN	1	>128	45 (32.61)
	AMK	2	4	14 (10.14)
	STR	8	>128	55 (39.86)
	SPE	32	>128	37 (26.81)
Quinolones	LEV	0.5	16	24 (17.39)
	ENR	0.5	16	22 (15.94)
	NAL	32	>1024	75 (54.35)
	CIP	0.5	>32	41 (29.71)
Tetracyclines	TIG	0.5	1	0
	DOX	8	32	64 (46.38)
	TET	8	>64	66 (47.83)
Polypeptides	COL	0.25	2	9 (6.52)
Sulfonamides	SXZ	>1024	>1024	138 (100)
Amide alcohols	CHL	4	>128	42 (30.43)
	FOS	16	256	16 (11.59)

Abbreviations: AMP, ampicillin; CTX, cefotaxime; CAZ, ceftazidime; CTF, cefotiofur; MER, meropenem; KAN, kanamycin; GEM, gentamicin; AMK, amikacin; STR, streptomycin; SPE, spectinomycin; LEV, levofloxacin; ENR, enrofloxacin; NAL, nalidixic acid; CIP, ciprofloxacin; TIG, tigacycline; DOX, doxycycline; TET, tetracycline; COL, colistin; SXZ, sulfisoxazole; CHL, chloramphenicol; FOS, fosfomycin. MIC_50_ = MIC at which growth was inhibited in 50% of isolates; MIC_90_ = MIC at which growth was inhibited in 90% of isolates.

## Data Availability

The data presented in the study are deposited in the NCBI repository, accession number PRJNA1117363.

## Supplementary Materials

The following supporting information can be downloaded at: https://www.mdpi.com/article/10.3390/antibiotics13100959/s1, Table S1: The ARGs’ primer sequences used for PCR; Table S2: Information on the 24 strains used for whole-genome sequencing.

## References

[1] Crump J.A., Luby S.P., Mintz E.D. (2004). The global burden of typhoid fever. Bull. World Health Organ..

[2] Bresee J.S., Marcus R., Venezia R.A., Keene W.E., Morse D., Thanassi M., Brunett P., Bulens S., Beard R.S., Dauphin L.A. (2012). The etiology of severe acute gastroenteritis among adults visiting emergency departments in the United States. J. Infect. Dis..

[3] Havelaar A.H., Kirk M.D., Torgerson P.R., Gibb H.J., Hald T., Lake R.J., Praet N., Bellinger D.C., De Silva N.R., Gargouri N. (2015). World Health Organization Global Estimates and Regional Comparisons of the Burden of Foodborne Disease in 2010. PLoS Med..

[4] Wang X.C., Biswas S., Paudyal N., Pan H., Li X.L., Fang W.H., Yue M. (2019). Antibiotic Resistance in Typhimurium Isolates Recovered from the Food Chain through National Antimicrobial Resistance Monitoring System between 1996 and 2016. Front. Microbiol..

[5] Zakaria Z., Hassan L., Ahmad N., Husin S.A., Ali R.M., Sharif Z., Sohaimi N.M., Garba B. (2021). Discerning the Antimicrobial Resistance, Virulence, and Phylogenetic Relatedness of *Salmonella* Isolates Across the Human, Poultry, and Food Materials Sources in Malaysia. Front. Microbiol..

[6] Voss-Rech D., Potter L., Vaz C.S., Pereira D.I., Sangioni L.A., Vargas A.C., de Avila Botton S. (2017). Antimicrobial Resistance in Nontyphoidal *Salmonella* Isolated from Human and Poultry-Related Samples in Brazil: 20-Year Meta-Analysis. Foodborne Pathog. Dis..

[7] Wang W., Zhao L., Hu Y., Dottorini T., Fanning S., Xu J., Li F. (2020). Epidemiological Study on Prevalence, Serovar Diversity, Multidrug Resistance, and CTX-M-Type Extended-Spectrum beta-Lactamases of *Salmonella* spp. from Patients with Diarrhea, Food of Animal Origin, and Pets in Several Provinces of China. Antimicrob. Agents Chemother..

[8] Centers for Disease C Prevention (2013). Incidence and trends of infection with pathogens transmitted commonly through food—Foodborne diseases active surveillance network, 10 U.S. sites, 1996-2012. MMWR Morb. Mortal. Wkly. Rep..

[9] Liu Q., Chen W., Elbediwi M., Pan H., Wang L., Zhou C., Zhao B., Xu X., Li D., Yan X. (2020). Characterization of *Salmonella* Resistome and Plasmidome in Pork Production System in Jiangsu, China. Front. Vet. Sci..

[10] Chu Y., Wang D., Hao W., Sun R., Sun J., Liu Y., Liao X. (2024). Prevalence, antibiotic resistance, virulence genes and molecular characteristics of *Salmonella* isolated from ducks and wild geese in China. Food Microbiol..

[11] Shen X.H., Yin L., Zhang A.Y., Zhao R.H., Yin D.D., Wang J.R., Dai Y., Hou H.Y., Pan X.C., Hu X.M. (2023). Prevalence and Characterization of Isolated from Chickens in Anhui, China. Pathogens.

[12] Barnett R. (2016). Typhoid fever. Lancet.

[13] Colin P. (2018). International Symposium on *Salmonella* and salmonellosis. Food Microbiol..

[14] Littman R.J. (2009). The plague of Athens: Epidemiology and paleopathology. Mt. Sinai J. Med..

[15] Medalla F., Gu W., Mahon B.E., Judd M., Folster J., Griffin P.M., Hoekstra R.M. (2016). Estimated Incidence of Antimicrobial Drug-Resistant Nontyphoidal *Salmonella* Infections, United States, 2004–2012. Emerg. Infect. Dis..

[16] Peng M., Salaheen S., Buchanan R.L., Biswas D. (2018). Alterations of *Salmonella* enterica Serovar Typhimurium Antibiotic Resistance under Environmental Pressure. Appl. Environ. Microbiol..

[17] Dai W., Zhang Y., Zhang J., Xue C., Yan J., Li X., Zheng X., Dong R., Bai J., Su Y. (2021). Analysis of antibiotic-induced drug resistance of *Salmonella* enteritidis and its biofilm formation mechanism. Bioengineered.

[18] Kagambega A., McMillan E.A., Bouda S.C., Hiott L.M., Ramadan H., Soro D.K., Sharma P., Gupta S.K., Barro N., Jackson C.R. (2022). Resistance Genes, Plasmids, Multilocus Sequence Typing (MLST), and Phenotypic Resistance of Non-Typhoidal *Salmonella* (NTS) Isolated from Slaughtered Chickens in Burkina Faso. Antibiotics.

[19] Yang J., Gao S.W., Chang Y.J., Su M.L., Xie Y.T., Sun S.H. (2019). Occurrence and Characterization of Isolated from Large-Scale Breeder Farms in Shandong Province, China. BioMed Res. Int..

[20] Zhao X., Gao Y., Ye C., Yang L., Wang T., Chang W. (2016). Prevalence and Characteristics of *Salmonella* Isolated from Free-Range Chickens in Shandong Province, China. BioMed Res. Int..

[21] Zhu Y., Lai H., Zou L., Yin S., Wang C., Han X., Xia X., Hu K., He L., Zhou K. (2017). Antimicrobial resistance and resistance genes in *Salmonella* strains isolated from broiler chickens along the slaughtering process in China. Int. J. Food Microbiol..

[22] Foley S.L., Lynne A.M., Nayak R. (2008). *Salmonella* challenges: Prevalence in swine and poultry and potential pathogenicity of such isolates. J. Anim. Sci..

[23] Kuang X.H., Hao H.H., Dai M.H., Wang Y.L., Ahmad I., Liu Z.L., Yuan Z.H. (2015). Serotypes and antimicrobial susceptibility of spp. isolated from farm animals in China. Front. Microbiol..

[24] Tang B., Elbediwi M., Nambiar R.B., Yang H., Lin J., Yue M. (2022). Genomic Characterization of Antimicrobial-Resistant *Salmonella* enterica in Duck, Chicken, and Pig Farms and Retail Markets in Eastern China. Microbiol. Spectr..

[25] Xu H.Y., Zhang W.B., Zhang K., Zhang Y., Wang Z.Y., Zhang W., Li Y., Li Q.C. (2021). Characterization of serotypes prevalent in asymptomatic people and patients. BMC Infect. Dis..

[26] Cui M.Q., Xie M.Y., Qu Z.N., Zhao S.J., Wang J.W., Wang Y., He T., Wang H.Y., Zuo Z.C., Wu C.M. (2016). Prevalence and antimicrobial resistance of isolated from an integrated broiler chicken supply chain in Qingdao, China. Food Control.

[27] Elsayed M.M., El-Basrey Y.F.H., El-Baz A.H., Dowidar H.A., Shami A., Al-Saeed F.A., Alsamghan A., Salem H.M., Alhazmi W.A., El-Tarabily K.A. (2024). Ecological prevalence, genetic diversity, and multidrug resistance of *Salmonella* enteritidis recovered from broiler and layer chicken farms. Poult. Sci..

[28] Liu C.X., Yao K.Y., Ren D.X., Xiao Y.P. (2022). Prevalence and characterization of from meat in slaughterhouses in Hangzhou, China. Int. J. Food Microbiol..

[29] Cai Y., Tao J., Jiao Y., Fei X., Zhou L., Wang Y., Zheng H., Pan Z., Jiao X. (2016). Phenotypic characteristics and genotypic correlation between *Salmonella* isolates from a slaughterhouse and retail markets in Yangzhou, China. Int. J. Food Microbiol..

[30] Dessie H.K., Bae D.H., Lee Y.J. (2013). Characterization of integrons and their cassettes in and isolates from poultry in Korea. Poult. Sci..

[31] Miranda J.M., Rodriguez J.A., Galan-Vidal C.A. (2009). Simultaneous determination of tetracyclines in poultry muscle by capillary zone electrophoresis. J. Chromatogr. A.

[32] Costa M.M., Cardo M., Soares P., Cara d’Anjo M., Leite A. (2022). Multi-Drug and beta-Lactam Resistance in Escherichia coli and Food-Borne Pathogens from Animals and Food in Portugal, 2014–2019. Antibiotics.

[33] Drauch V., Kornschober C., Palmieri N., Hess M., Hess C. (2021). Infection dynamics of Infantis strains displaying different genetic backgrounds—With or without pESI-like plasmid—Vary considerably. Emerg. Microbes Infect..

[34] Song Y., Wang F.K., Liu Y., Song Y.Y., Zhang L., Zhang F.Y., Gu X.X., Sun S.H. (2020). Occurrence and Characterization of Isolated from Chicken Breeder Flocks in Nine Chinese Provinces. Front. Vet. Sci..

[35] Zhao L., Liu G., Tang W., Song X., Zhao X., Wang C., Li Y., Zou M. (2023). Antimicrobial resistance and genomic characteristics of *Salmonella* from broilers in Shandong Province. Front. Vet. Sci..

[36] Li Y., Kang X.M., Ed-Dra A., Zhou X., Jia C.H., Müller A., Liu Y.Q., Kehrenberg C., Yue M. (2022). Genome-Based Assessment of Antimicrobial Resistance and Virulence Potential of Isolates of Non-Pullorum/Gallinarum Serovars Recovered from Dead Poultry in China. Microbiol. Spectr..

[37] Yu X., Zhu H., Bo Y., Li Y., Zhang Y., Liu Y., Zhang J., Jiang L., Chen G., Zhang X. (2021). Prevalence and antimicrobial resistance of *Salmonella* enterica subspecies enterica serovar Enteritidis isolated from broiler chickens in Shandong Province, China, 2013–2018. Poult. Sci..

[38] Zhao X., Hu M., Zhang Q., Zhao C., Zhang Y., Li L., Qi J., Luo Y., Zhou D., Liu Y. (2020). Characterization of integrons and antimicrobial resistance in *Salmonella* from broilers in Shandong, China. Poult. Sci..

[39] Yang F., Jiang Y.G., Yang L.H., Qin J.X., Guo M.Q., Lu Y.X., Chen H.Y., Zhuang Y., Zhang J.H., Zhang H. (2018). Molecular and Conventional Analysis of Acute Diarrheal Isolates Identifies Epidemiological Trends, Antibiotic Resistance and Virulence Profiles of Common Enteropathogens in Shanghai. Front. Microbiol..

[40] Li R.C., Lai J., Wang Y., Liu S.L., Li Y., Liu K.Y., Shen J.Z., Wu C.M. (2013). Prevalence and characterization of species isolated from pigs, ducks and chickens in Sichuan Province, China. Int. J. Food Microbiol..

[41] Liu W.B., Chen J., Huang Y.Y., Liu B., Shi X.M. (2010). Serotype, genotype, and antimicrobial susceptibility profiles of *Salmonella* from chicken farms in Shanghai. J. Food Prot..

[42] Nasrin S., Fuche F.J., Sears K.T., Jones J.A., Levine M.M., Simon R., Tennant S.M. (2021). Refinement of a Live Attenuated *Salmonella enterica* Serovar Newport Vaccine with Improved Safety. Vaccines.

[43] Sun L., Zhang H.X., Chen J., Chen L.L., Qi X.J., Zhang R.H. (2021). Epidemiology of Foodborne Disease Outbreaks Caused by Nontyphoidal in Zhejiang Province, China, 2010–2019. Foodborne Pathog. Dis..

[44] Ansari-Lari M., Hosseinzadeh S., Manzari M., Khaledian S. (2022). Survey of *Salmonella* in commercial broiler farms in Shiraz, southern Iran. Prev. Vet. Med..

[45] Ramtahal M.A., Amoako D.G., Akebe A.L.K., Somboro A.M., Bester L.A., Essack S.Y. (2022). A Public Health Insight into in Poultry in Africa: A Review of the Past Decade: 2010–2020. Microb. Drug Resist..

[46] Baker M., Zhang X., Maciel-Guerra A., Babaarslan K., Dong Y., Wang W., Hu Y., Renney D., Liu L., Li H. (2024). Convergence of resistance and evolutionary responses in *Escherichia coli* and *Salmonella enterica* co-inhabiting chicken farms in China. Nat. Commun..

[47] Gu G.Y., Strawn L.K., Zheng J., Reed E.A., Rideout S.L. (2019). Diversity and Dynamics of in Water Sources, Poultry Litters, and Field Soils Amended With Poultry Litter in a Major Agricultural Area of Virginia. Front. Microbiol..

[48] Truitt L.N., Vazquez K.M., Pfuntner R.C., Rideout S.L., Havelaar A.H., Strawn L.K. (2018). Microbial Quality of Agricultural Water Used in Produce Preharvest Production on the Eastern Shore of Virginia. J. Food Prot..

[49] Willmann M., El-Hadidi M., Huson D.H., Schütz M., Weidenmaier C., Autenrieth I.B., Peter S. (2015). Antibiotic Selection Pressure Determination through Sequence-Based Metagenomics. Antimicrob. Agents Chemother..

[50] Dantas G., Sommer M.O.A. (2012). Context matters—The complex interplay between resistome genotypes and resistance phenotypes. Curr. Opin. Microbiol..

[51] Gomi R., Matsuda T., Matsumura Y., Yamamoto M., Tanaka M., Ichiyama S., Yoneda M. (2017). Whole-Genome Analysis of Antimicrobial-Resistant and Extraintestinal Pathogenic in River Water. Appl. Environ. Microbiol..

[52] Kosikowska U., Stec J., Andrzejczuk S., Mendrycka M., Pietras-Ozga D., Stepien-Pysniak D. (2022). Plasmid-Mediated Fluoroquinolone Resistance Genes in Quinolone-Susceptible spp. Phenotypes Isolated from Recreational Surface Freshwater Reservoir. Front. Cell. Infect. Microbiol..

[53] Liu G., Olsen J.E., Thomsen L.E. (2019). Identification of Genes Essential for Antibiotic-Induced Up-Regulation of Plasmid-Transfer-Genes in Cephalosporin Resistant. Front. Microbiol..

[54] Hinnebusch B.J., Rosso M.L., Schwan T.G., Carniel E. (2002). High-frequency conjugative transfer of antibiotic resistance genes to Yersinia pestis in the flea midgut. Mol. Microbiol..

[55] Sobisch L.Y., Rogowski K.M., Fuchs J., Schmieder W., Vaishampayan A., Oles P., Novikova N., Grohmann E. (2019). Biofilm Forming Antibiotic Resistant Gram-Positive Pathogens Isolated from Surfaces on the International Space Station. Front. Microbiol..

[56] Zhang Z., Wang Y., Chen B., Lei C., Yu Y., Xu N., Zhang Q., Wang T., Gao W., Lu T. (2022). Xenobiotic pollution affects transcription of antibiotic resistance and virulence factors in aquatic microcosms. Environ. Pollut..

[57] Gong J.S., Zeng X.M., Zhang P., Zhang D., Wang C.M., Lin J. (2019). Characterization of the emerging multidrug-resistant serovar Indiana strains in China. Emerg. Microbes Infect..

[58] Wang X.F., Wang T., Guo M.J., Zhang C.C., Bo Z.Y., Wu Y.T., Chao G.X. (2022). The large plasmid carried class 1 integrons mediated multidrug resistance of foodborne Indiana. Front. Microbiol..

[59] Ghosh S., Biswas S., Mukherjee S., Pal A., Saxena A., Sundar S., Dujardin J.C., Das S., Roy S., Mukhopadhyay R. (2021). A Novel Bioimpedance-Based Detection of Miltefosine Susceptibility Among Clinical Isolates of the Indian Subcontinent Exhibiting Resistance to Multiple Drugs. Front. Cell. Infect. Microbiol..

[60] McLellan H., Harvey S.E., Steinbrenner J., Armstrong M.R., He Q., Clewes R., Pritchard L., Wang W., Wang S., Nussbaumer T. (2022). Exploiting breakdown in nonhost effector-target interactions to boost host disease resistance. Proc. Natl. Acad. Sci. USA.

[61] Cossi M.V.C., Polveiro R.C., Yamatogi R.S., Camargo A.C., Nero L.A. (2024). Multi-locus sequence typing, antimicrobials resistance and virulence profiles of *Salmonella enterica* isolated from bovine carcasses in Minas Gerais state, Brazil. Braz. J. Microbiol..

[62] Rahn K., De Grandis S.A., Clarke R.C., McEwen S.A., Galan J.E., Ginocchio C., Curtiss R., Gyles C.L. (1992). Amplification of an invA gene sequence of *Salmonella typhimurium* by polymerase chain reaction as a specific method of detection of *Salmonella*. Mol. Cell. Probes.

[63] Hong Y., Liu T., Lee M.D., Hofacre C.L., Maier M., White D.G., Ayers S., Wang L., Berghaus R., Maurer J.J. (2008). Rapid screening of *Salmonella* enterica serovars Enteritidis, Hadar, Heidelberg and Typhimurium using a serologically-correlative allelotyping PCR targeting the O and H antigen alleles. BMC Microbiol..

[64] The Clinical and Laboratory Standards Institute (CLSI) (2015). Performance Standards for Antimicrobial Susceptibility Testing; Twenty-Fifth Informational Supplement.

[65] Letunic I., Bork P. (2021). Interactive Tree Of Life (iTOL) v5: An online tool for phylogenetic tree display and annotation. Nucleic Acids Res..

