# Supplementary material for: Serotype Distribution and Antimicrobial Resistance of Salmonella Isolates from Poultry Sources in China
Source: Antibiotics (Basel). 2024 Oct 11;13(10):959. doi: 10.3390/antibiotics13100959 (PMC11503990; doi:10.3390/antibiotics13100959)
Supplement: Supplementary file 1 [file antibiotics-13-00959-s001.zip › antibiotics-3222491-supplementary.pdf]

Table S1. The ARGs' primer sequences used for PCR.

Category	Target gene	Primer sequences (5'-3')	Size (bp)
Aminoglycosides	*aac*	for: 5'-CTGATGTCGATGGGCAGGAC-3'<br>rev: 5'-CATCCGTCAGATGGTCACCG-3'	423
	aph(3')-Ia	for: 5'-TAGAGTCTGCGCCATGAGTG-3'<br>rev: 5'-GCGGATCGAGATGGAGGTAG-3'	818
	aph(3'')-Ib	for: 5'-GAAAATCGCACCTGCTTCCC-3'<br>rev: 5'-CAAGTTGCTGCCCCATTGAC-3'	218
	aph(6)-Id	for: 5'-GGGACTCCTGCAATCGTCAA-3'<br>rev: 5'-ATATAGCCCACGCAGTTCCG-3'	408
	aadA1	for: 5'-TATCAGAGGTAGTTGGCGTCAT-3'<br>rev: 5'-GCGAGTTCCATAGCGTTAAGG-3'	489
	aadA2	for: 5'-CTGCGCATTTTGGTTCCCTC-3'<br>rev: 5'-GCCAGCAACGTTGATTCCAG-3'	469
β-lactams	*$bla_{CTX\text{-}M\text{-}55}$*	for: 5'-ATGTGCAGYACCAGTAARGTKATGGC-3'<br>rev: 5'-TGGGTRAARTARGTSACCAGAAYCAGCGG-3'	593
	$bla_{TEM\text{-}1B}$	for: 5'-GTCGCCGCATACACTATT-3'<br>rev: 5'-CGCCTCCATCCAGTCTAT-3'	365
Chloramphenicol	*floR*	for: 5'-CTGAGGGTGTCGTCATCTAC-3'<br>rev: 5'-GCTCCGACAATGCTGACTAT-3'	673
Folate pathway	*dfrA12*	for: 5'-CTGCGCATTTTGGTTCCCTC-3'<br>rev: 5'-GCCAGCAACGTTGATTCCAG-3'	469
Fosfomycins	*fosA3*	for: 5'-GTAAAACGCCCCCTCAGGAA-3'<br>rev: 5'-ACGCTCAGAAGCTCAACGAA-3'	350
Macrolides	*mphA*	for: 5'-TCGAAGACTCGACTGCGATG-3'<br>rev: 5'-TAGAGATCGCCATGCACCAC-3'	316
	mphE	for: 5'-TCGTGATGGCATGAGGGAAC-3'<br>rev: 5'-GCCCAGCAAAATCAATCGCT-3'	539

	msrE	for: 5’-CCAACCAGCCACCTTGATCT-3’<br>rev: 5’-TGCACGAATTTCCGCAACTG-3’	795
Tetracyclines	*tetA*	for: 5’-CATCGCAGACAACGTGAACC-3’<br>rev: 5’-CACGGTGGTTTTGTCTGCTC-3’	316
	gyrA	for: 5’-AGTCCTATCTCGACTACGCGAT-3’<br>rev: 5’-AGTCGACGGTTTCCTTTTCCAG-3’	341
	gyrB	for: 5’-TGCGGTGGAACAGGAGATGGGCAAGTAC-3’<br>rev: 5’-CTGGCGGAAGAAGAAGGTCAACAGCAGGGT-3’	697
Quinolones	*qnrD*	for: 5’-CGAGATCAATTTACGGGGAATA-3’<br>rev: 5’-AACAAGCTGAAGCGCCTG-3’	644
	qnrB	for: 5’-GGMATHGAAATTCGCCACTG-3’<br>rev: 5’-TTTGCYGYYCGCCAGTCGAA-3’	264
	OqxB	for: 5’-CTCGAACGGCTATCAGGGAC-3’<br>rev: 5’-GCCTGGTAAGTCGAGATCGG-3’	585
	sul1	for: 5’-TGGTGACGGTGTTCGGCATTC-3’<br>rev: 5’-GCGAGGGTTTCCGAGAAGGTG-3’	798
Sulfonamides	*sul2*	for: 5’-TCAACATAACCTCGGACAGT-3’<br>rev: 5’-GATGAAGTCAGCTCCACCT-3’	707
	sul3	for: 5’-CGTAAATATAACCACCGAT-3’<br>rev: 5’-CCAAGCCTGAATAAATCTCA-3’	326
Polymyxin	*mcr*	for: 5’-ATCGTATCGCTATGTGCTAA-3’<br>rev: 5’-CAGGCTTGGTTGCTTGTA-3’	488

Table S2. Information on the 24 strains used for whole-genome sequencing.

Strain	Location	Source	Serotype	Antimicrobial resistance
JSL3	Jiangsu	Layer	Enteritidis	AMP-NAL-SXZ
JSL7	Jiangsu	Layer	Enteritidis	STR-NAL-SXZ
JSM1	Jiangsu	Broiler	Indiana	SPE-NAL-DOX-TET-CHL-SXZ
XJL1	Xinjiang	Layer	N/A	NAL-SXZ
XJL2	Xinjiang	Layer	Typhimurium	STR-NAL-DOX-TET-SXZ
SDM1	Shandong	Broiler	Indiana	CTX-CAZ-CTF-KAN-GEN-STR-SPE-LEV-ENR-NAL-CIP-DOX-TET-CHL-SXZ
SDM3	Shandong	Broiler	Indiana	CTX-CAZ-CTF-KAN-GEN-STR-SPE-LEV-ENR-NAL-CIP-DOX-TET-CHL-SXZ
SDM4	Shandong	Broiler	Indiana	AMP-CTX-CAZ-CTF-KAN-GEN-STR-SPE-LEV-ENR-NAL-CIP-DOX-TET-CHL-SXZ
SDM7	Shandong	Broiler	Indiana	AMP-CTX-CAZ-CTF-KAN-GEN-STR-SPE-LEV-ENR-NAL-CIP-DOX-TET-CHL-SXZ
SDM8	Shandong	Broiler	Indiana	AMP-CTX-CAZ-CTF-KAN-GEN-STR-SPE-LEV-ENR-NAL-CIP-DOX-TET-CHL-SXZ
SDM9	Shandong	Broiler	Indiana	AMP-CTX-CAZ-CTF-KAN-GEN-STR-SPE-LEV-ENR-CIP-DOX-TET-CHL-SXZ
SDM11	Shandong	Broiler	Indiana	AMP-CTX-CAZ-CTF-KAN-GEN-STR-SPE-LEV-ENR-CIP-DOX-TET-CHL-SXZ
SDM18	Shandong	Broiler	Newport	AMP-CTX-CAZ-CTF-KAN-GEN-AMK-STR-CIP-DOX-TET-CHL-SXZ
AHL21	Anhui	Layer	N/A	SXZ
AHM34	Anhui	Broiler	London	AMP-GEN-STR-NAL-CIP-DOX-TET-SXZ
AHM8	Anhui	Broiler	Indiana	CTX-CAZ-CTF-KAN-GEN-AMK-SPE-LEV-ENR-NAL-CIP-DOX-TET-CHL-SXZ
AHM39	Anhui	Broiler	Indiana	AMP-CTX-CAZ-CTF-KAN-GEN-AMK-SPE-LEV-ENR-NAL-CIP-DOX-TET-CHL-SXZ
AHM40	Anhui	Broiler	Indiana	AMP-CTX-CAZ-CTF-KAN-GEN-AMK-STR-SPE-LEV-ENR-NAL-CIP-DOX-TET-CHL-SXZ
AHM41	Anhui	Broiler	Indiana	AMP-CTX-CAZ-CTF-KAN-GEN-AMK-STR-SPE-LEV-ENR-NAL-CIP-DOX-TET-CHL-SXZ
AHM43	Anhui	Broiler	Indiana	AMP-CTX-CAZ-CTF-KAN-GEN-AMK-SPE-LEV-ENR-NAL-CIP-DOX-TET-CHL-SXZ
AHM45	Anhui	Broiler	Indiana	AMP-CTX-CAZ-CTF-KAN-GEN-AMK-SPE-LEV-ENR-NAL-CIP-DOX-TET-CHL-SXZ

AHM47	Anhui	Broiler	Indiana	AMP-CTX-CAZ-CTF-KAN-GEN-AMK-STR-SPE-LEV-ENR-NAL-CIP-DOX-TET-CHL-SXZ
ZJM2	Zhejiang	Broiler	Indiana	NAL-DOX-TET-CHLSXZ
ZJM3	Zhejiang	Broiler	Indiana	KAN-NAL-DOX-TET-CHL-SXZ
ZJL2	Zhejiang	Layer	London	AMP-GEN-STR-NAL-CIP-DOX-TET-SXZ
